# Nutrient intakes and nutritional biomarkers in pregnant adolescents: a systematic review of studies in developed countries

**DOI:** 10.1186/s12884-016-1059-9

**Published:** 2016-09-15

**Authors:** Katie Marvin-Dowle, Victoria Jane Burley, Hora Soltani

**Affiliations:** 1 Centre for Health and Social Care Research, Sheffield Hallam University, Collegiate Crescent, Sheffield, S10 2BP UK; 2 School of Food Sciences and Nutrition, University of Leeds, Leeds, LS2 9JT UK

**Keywords:** Adolescent, Pregnancy, Nutrition, Systematic review

## Abstract

**Background:**

Babies born to adolescent mothers have been shown to have poorer outcomes compared to those born to adults. Nutritional status may have an important role to play in improving the health of pregnant adolescents; however there is a lack of evidence regarding the adequacy of adolescent diets during pregnancy. This systematic review aims to examine what is known about the nutritional status of adolescent pregnant women.

**Methods:**

A systematic search of the literature identified 21 studies which met the inclusion criteria for the review. Primary research papers using any methods were included where they were published in English between January 1995 and May 2015 and included measurements of nutrient intakes or biological markers of nutritional status in pregnant women aged 11–19 years. Individual study data was first summarised narratively before study means were pooled to give an estimate of nutritional status in the population.

**Results:**

The results show that individual studies reported intakes of energy, fibre and a number of key micronutrients which were below recommended levels. Biological markers of iron and selenium status also showed cause for concern. Pooled analysis of individual means as a percentage of UK Dietary Reference Intakes showed intakes of vitamin D (34.8 % CI 0–83.1) to be significantly below recommendations (*p* = 0.05). Serum selenium levels were also found to be low (61.8 μg/L, CI 39–84).

**Conclusions:**

This review has identified a number of areas where the nutritional status of pregnant adolescents is sub-optimal, which may have implications for the health of adolescent mothers and their babies. It was not however possible to examine the impact of supplement use or socio-demographic characteristics which limits the interpretation these results. Further work is needed to establish the characteristics of those most at risk within this population, how this differs from adult pregnant women and the role of supplementation in achieving adequate nutrition.

**Electronic supplementary material:**

The online version of this article (doi:10.1186/s12884-016-1059-9) contains supplementary material, which is available to authorized users.

## Background

Pregnancy during adolescence is often viewed as a social problem with women who have a child during the teenage years being more likely to suffer social isolation, poverty, lower levels of educational achievement and be unemployed or work in low paid jobs [1]. Rates of teenage conceptions both in the UK and internationally have reduced over recent years; however there are still a significant number of young women having pregnancies and giving birth at a young age. The rate of deliveries to young women aged 15–19 in the UK in 2012 was the highest in the European Union at 19.7 births per 1,000 females in the age group. This does however represent a reduction of more than a quarter (26.8 %) in the UK since 2004. The birth rates to young women in other developed countries have followed a similar pattern of decline, yet rates remain relatively high in the United States (29.4), New Zealand (24.9) and Australia (16.1) [2].

As well as the potential for adverse social outcomes associated with adolescent pregnancies there is evidence to suggest that health outcomes may be less favourable for younger mothers. A systematic review [3] aiming to assess the relationship between early first childbirth and increased risk of poor pregnancy outcomes found that very young maternal age (<15 years or less than 2 years after menarche) had a negative effect on both maternal and foetal growth and infant survival. It is suggested that young women who are still themselves growing may compete with the foetus for nutrients, which may in turn impair foetal growth and result in low birth weight babies or babies who are small for their gestational age. The review also found a moderate relationship between young maternal age and anaemia, premature birth and neonatal mortality.

It has long been established that good pregnancy nutrition has an important influence on birth outcomes, foetal growth and infant survival [4]. While specific nutritional issues may have changed since this early work, it is still maintained that mothers need to consume an adequate, yet not excessive diet in order to optimise pregnancy and birth outcomes [5]. Quantification of dietary adequacy in populations is difficult because individuals will have differing nutrient needs. This is especially true during phases of growth and physical change such as adolescence. However, the use of dietary reference intakes to estimate the adequacy of nutritional intakes has been established as acceptable [6] where the appropriate values for the age, sex and, in the case of pregnant women, stage of pregnancy are used. Evidence also suggests that nutritional needs change during the course of pregnancy with requirements for energy and several micronutrients increasing as pregnancy progresses [7].

Dietary habits of adolescent girls are often poorer than that of older women. The latest results of the UK National Diet and Nutrition Survey [8] showed that girls aged 11–18 years consumed 2.7 portions of fruit and vegetables per day compared to 4.1 portions in women aged 19–64, and adolescent girls also had some of the highest intakes of sugar-sweetened beverages within this dataset. A higher proportion of adolescent girls also had intakes of key vitamins and minerals below the lower reference nutrient intake level than adult women, including vitamin A, riboflavin, vitamin B_12_, folate, iron, calcium, magnesium, potassium, zinc and iodine. Dietary patterns across highly developed countries have been shown to have substantial similarities [9], while the same cannot be said for less developed regions.

While the evidence presented above suggests that adolescent girls often have a poorer diet than adult women in the general population this may not also necessarily be the case in those who are pregnant. Two systematic reviews have previously been conducted [10, 11] which explored nutritional intake and biochemical markers in pregnant adolescents living in developed countries. It was acknowledged in these reviews that there was a lack of good quality evidence in relation to these topics. However the author concluded that there was some consensus in the available literature that pregnant adolescents had intakes of energy, iron, folate, calcium, vitamin E and magnesium which were below the dietary recommended intakes. The review of biochemical markers reported that indicators of anaemia and iron status were compromised in this population; however no further conclusions could be drawn from the limited available evidence. It is therefore important that the most recent evidence relating to the nutritional intake and status of pregnant adolescents is examined in order to establish what the particular issues may be for this group. The aim of his systematic review was therefore to investigate the nutritional status of pregnant adolescents living in developed countries.

## Methods

### Search strategy

The search strategy was developed using search terms detailed in Table 1 and applied across nine key electronic databases (AMED, ASSIA, CINAHL, Child Development and Adolescent Studies, Cochrane Library, Health Source: Nursing, Maternity and Infant Care, MEDLINE and MEDLINE in Process, SCOPUS). Reference lists of identified papers were hand searched, and reference and citation functions were used where available.

**Table 1:** Search terms

Theme	Nutritional Intake	Pregnancy	Age	Nutritional Status
Search Terms	nutrient	pregnan*	adolescen*	biomarker*
	nutrition*	gestatio*	teen	iron
	diet*	matern*	teenage*	folate
	eat*	mother*	youth	calcium
	food	gravid*		anaemi*
	nutrition assessment (MH)	Pregnancy in adolescence (MH)		anemi*
	Food habits (MH)			Biological markers (MH)
	Dietary surveys (MH)			

* indicates truncation of search tearm

**Fig. 1:**
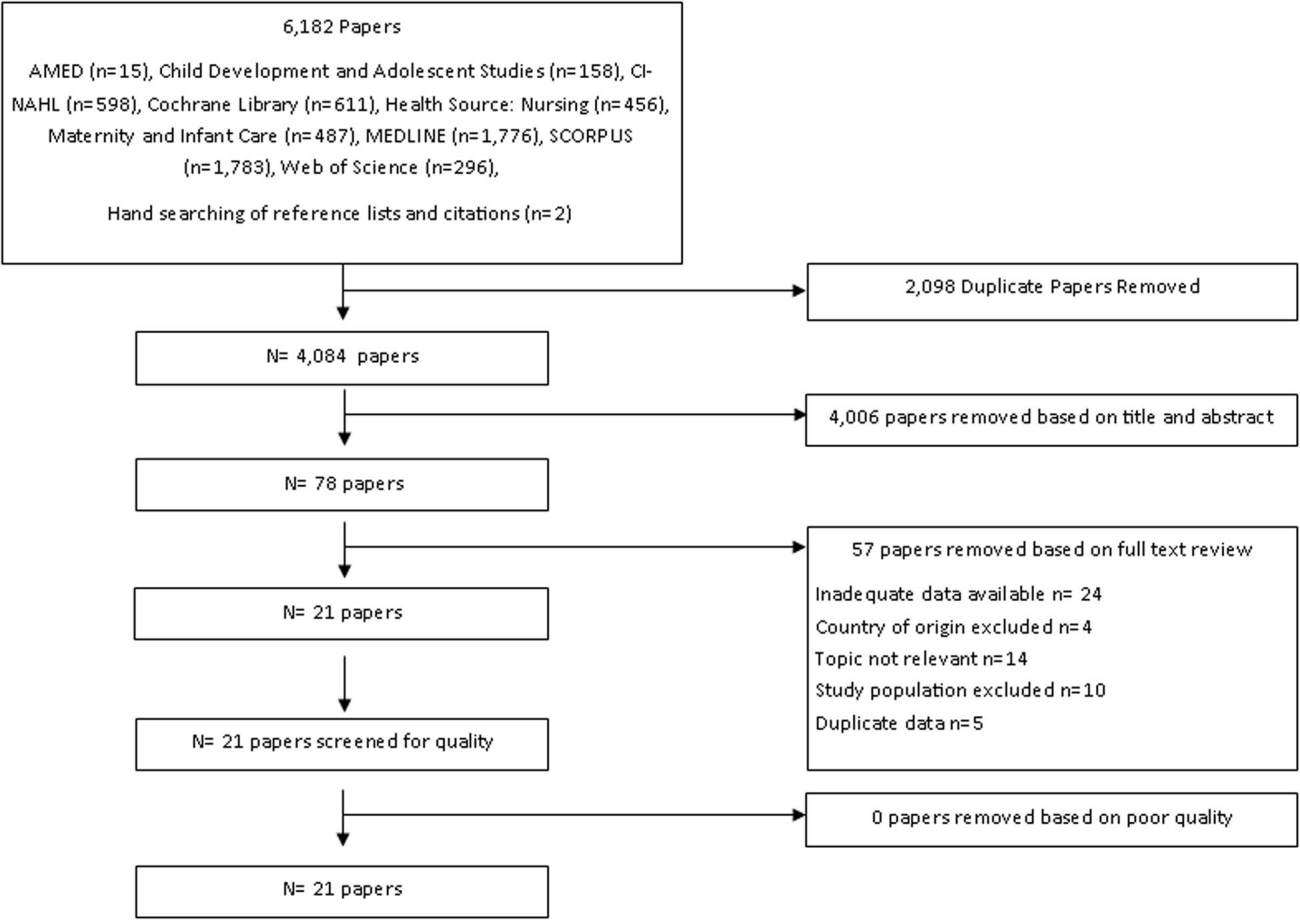
Protocol for systematic review

Table 1 search terms The main stages of the review including the number of references identified at each stage are illustrated in Fig. 1.

### Inclusion criteria

Studies were limited to primary research papers using any methods published in English between 1995 and May 2015.

Studies were included where they provided data regarding either the nutritional intake or a biological marker of nutrient status of adolescents aged 11–19 years at any stage of pregnancy, from countries considered as having very high levels of human development by the United Nations Human Development Index [12]. This index has been selected as it provides a multidimensional model incorporating not only wealth but also health and education, and so provides a more reliable basis for assuming some commonality between countries of origin of the included studies. The definition of adolescence has been chosen to correspond with the World Health Organisation Growth Index [13].

### Quality appraisal

The identified papers were assessed for risk of bias using the Critical Appraisal and Skills Program (CASP) checklist for systematic reviews [14] which was adapted to accommodate cross-sectional studies. Aspects of the studies giving an indication of methodological and interpretive rigor (e.g. research design, clear statement of research aims, recruitment of participants, consideration of confounding factors and reporting of results) were graded as either ‘good’ (+), ‘adequate or unclear’ (−/+) or poor (−), studies were then given an overall grade for quality.

### Data extraction

Included studies were grouped depending on whether the study examined nutrient intake, biological markers of nutritional status or both. Information from the included studies was entered in to data extraction sheets using Microsoft Excel, one each for nutrient intakes and biological markers, and checked by a second reviewer.

### Data synthesis

Reported data were first tabulated to explore patterns across the included studies and described narratively. Where 95 % confidence intervals were not reported they were calculated for the mean and used to assess the extent to which the study mean differed from the relevant reference value. Where the lower confidence limit was above the reference value the study mean was considered to be significantly higher, where the upper confidence limit was below the reference value the study mean was considered significantly lower, otherwise no significant difference was reported.

Analysis of micronutrient intakes was undertaken by comparing the reported data from included studies with the UK reference nutrient intake (RNI) [15] and US recommended daily allowance (RDA) [16] where available. Analysis has been undertaken using both UK and US thresholds as, while the majority of studies were undertaken in the USA, this review is also concerned with applying the results to the UK context. Energy intake was calculated by taking the mean of the single year of age estimated average requirements (EAR) for young women aged 11–19 with an increment applied in the third trimester; a population level EAR is only available in the UK [17]. For macronutrients an estimate of the percentage contribution to energy, if not provided, was calculated from the mean macronutrient intake and the mean energy intake.

In the case of biological markers, minimum thresholds for nutrient deficiency provided by WHO, UK and US authorities (where available) have been compared with reported study data. Nationally or internationally recognised cut offs for deficiency are not currently available for zinc, selenium, copper, magnesium and phosphorous, therefore these elements have been compared with suggested thresholds in academic literature [18–22].

Where data were available from two or more studies for a single nutrient or biological marker a pooled mean was calculated and weighted by the number of participants in each study. Mean measures of micronutrient and energy intake were expressed as a percentage of dietary reference values to allow for comparisons across different nutrients.

Sub-group analyses were performed by country of origin (USA only and UK only), stage of pregnancy (first, second and third trimester and reported average over the pregnancy) and age of adolescents (15 years and under and 16–19 years).

## Results

A total of 4,084 unique papers were identified from the search of the literature with 78 studies remaining after title and abstract screening. Following examination of the full text of these papers a total of 21 papers were identified that met the review inclusion criteria. Details of the excluded studies are given in Additional file 1: Table S1. In brief, the main reasons for exclusion were not reporting appropriate data and the study population not meeting the inclusion criteria for age or pregnancy.

No studies were excluded for reasons of poor quality; after quality assessment 16 of the included studies were considered to be of good quality while the remaining five studies were of a satisfactory standard (Table 2).

**Table 2:** Characteristics of included studies

Study Information				Participants				Supplements			Measurement		Quality
Study	Country	Study design	Study groups	Number	Age	Inclusion criteria	Exclusion criteria	Supplement type and dose	Number with available data	Estimated compliance: N (%)	Data collection method (s)	Time period(s)	Quality rating
Baker et.al (2009) [23]	UK	Cross-sectional study	NA	500	14–18	Singleton pregnancy, age 14–18, gestational age < =20 weeks	Inability to provide informed consent, preeclampsia, clotting disorders, HIV/AIDS, haemoglobinopathies, diabetes, renal disease, hypertension, multiple gestations, history of 3= > previous miscarriages	Preconception folic acid	498	34 (6.9)	24 h recall (multiple)	Third trimester	+
								Folic acid in early pregnancy	498	220 (44.2)			
								Iron in early pregnancy	498	39 (7.8)			
								Multivitamins in early pregnancy	498	19 (3.8)			
								Folic acid only in 3rd trimester	290	5 (1.7)			
								Iron and folic acid in 3rd trimester	290	13 (4.5)			
								Iron only in 3rd trimester	290	42 (14.5)			
								Multivitamins in 3rd trimester	290	7 (2.4)			
Castillo-Duran et.al (2001) [43]	Chile	RCT	Zinc supplemented	249	16.4 (mean)	Beginning prenatal visits before 20 weeks gestation, aged <19 at estimated due date	Adolescents whose background included chronic diseases, drug abuse, mental retardation, illiteracy or pregnancy due to incest or rape	20 mg Zinc sulphate daily	Intervention group - 249	At least 50 % compliance	24 h recall (multiple)	Second and third trimester	+
			Placebo	258				40 mg iron sulphate	All participants - 507				
Chan et.al (2006) [24]	USA	RCT	Control group, 2 intervention groups excluded from review*	23	15–17	Enrolled before 20 weeks gestation	Hypertension, diabetes, renal or liver disease, alcohol or tobacco use, using medicines effecting Ca metabolism	No additional supplements reported			Food diary (weighing not mentioned)	Second and third trimester	−/+
Chang et.al (2003) [37]	USA	Retrospective chart review	NA	918	12–17	Self-reported racial group as African-American; singleton pregnancy	Birth results not available due to abortion, miscarriage or transfer of care	90 mg carbonyl iron daily plus additional up to 120 mg for those classified as anaemic	918	Not reported	Not reported	Second and third trimester	+
Dawson et.al (2000) [35]	USA	RCT	One-A-Day without Iron	20	16–20	Aged 16–20, less than 16 weeks gestation, no iron supplementation for previous 30 days	Hypertension; diabetes; other medical problems; haemoglobin <11 g/dL; haematocrit <30 %	Vitamin A 5000 IU, vitamin D 400 IU, vitamin E 10 mg, vitamin C 60 mg, folic acid 0.4 mg, thiamine 1.5 mg, riboflavin 1.7 mg, niacin 20 mg, pyridoxine 2 mg, vitamin B- 6 mg, pantothenic acid 10 mg	20	Not reported	Venous blood sample	Second and third trimester and delivery	−/+
			One-A-Day with Iron	20				18 mg Iron, Vitamin A 5000 IU, vitamin D 400 IU, vitamin E 10 mg, vitamin C 60 mg, folic acid 0.4 mg, thiamine 1.5 mg, riboflavin 1.7 mg, niacin 20 mg, pyridoxine 2 mg, vitamin B- 6 mg, pantothenic acid 10 mg	20				
Derbyshire (2009) [39]	UK	Cross-sectional study	NA	20	15–19	Attending antenatal classes or community clinics	Incomplete diary	None reported			Food diary unweighed	Third trimester	+
Gadowsky et.al (1995) [42]	Canada	Cross-sectional study	NA	58	14–19	Not reported	Not reported	Folic Acid Mean 479 μg/day	58	82 %	Venous blood sample	Third trimester	+
								Elemental Iron Mean 31.5 mg/day					
								Cyanocobalamin (B12) Mean 2.24 μg/d					
Giddens et.al (2000) [27]	USA	RCT (subset from a larger study)	NA	59	13–18	Singleton pregnancies, between 13 and 19 weeks gestation	Not reported	Reported that any contribution from supplements was not included in analysis			Food diary weighed	Mean over pregnancy	−/+
Ginde et.al (2010) [32]	USA	Secondary analysis of cross sectional survey	NA	84	13–19	Not reported	Not reported	Some participants taking vitamin D supplements	Not reported	Not reported	Venous blood sample	Mean over pregnancy	+
Gutierrez et.al (1999) [28]	USA	Cross-sectional study	NA	46	13–18	Self-identified ethnicity as Mexican American, primigravida, 13–18 years of age	History of miscarriage or health problems, involved in competitive athletic performances or heavy exercise, planned to move away during study period	None reported			24 h recall (single)	Second and third trimester	+
Iannotti et.al (2005) [36]	USA	Cross-sectional study	NA	80	13–18	Self-reported racial group as African-American; singleton pregnancy	Not reported	None reported			10 ml Venous blood sample	Second and third trimesters	+
Job et.al (1995) [41]	Australia	Cross-sectional study	NA	35	13–19	Not reported	Not reported	None reported			24 h recall	Mean over pregnancy	+
Lee et.al (2013) [25]	USA	Cross-sectional study	NA	156	13–18	Age 18 or under, carrying a single fetus, 12–30 weeks gestation at recruitment	Not reported	Reported that any contribution from supplements was not included in analysis			24 h recall (multiple)	<23 weeks gestation, 23–30 weeks gestation and Mean over pregnancy	+
McGuire et.al. (2010) [31]	USA	Cross-sectional study	NA	80	Under 18 (mean 16.5)	Self-reported ethnic group as African American, singleton pregnancy	Not reported	Routinely prescribed prenatal supplements containing 400 IU vitamin D		Data not available	Venous blood sample	Second and third trimester	+
Meier et.al (2002) [34]	USA	RCT	Iron supplemented	20	15–18	Not reported	Iron deficiency anaemia at recruitment	60 mg elemental iron & 1 mg folic acid	20		Venous blood sample	Second and third trimesters	+
			Placebo	17				1 mg folic acid	17				
Mistry et.al (2014) [40]	UK	Cross-sectional study	Appropriate for gestational age babies	107	14–18	Not reported	Inability to provide informed consent, pre-eclampsia, clotting disorders, HIV/AIDS, Haemoglobinpathies, diabetes, renal disease, hypertension, multiple pregnancy and previous miscarriage	None reported			30 ml venous blood sample	Third trimester	+
			Small for gestational age babies	9									
O’Brien et.al (2003) [33]	USA	Cross-sectional study	NA	23	Mean 16.5	First, singleton pregnancies; no medical problems; no medications known to influence calcium metabolism; non-smokers; no history of drug or alcohol abuse	Not reported	Prenatal supplement including 5 mmol Ca	23	39 %	Not reported	Third trimester	+
Pobocik et.al (2003) [38]	Guam (USA Teritory)	Cross-sectional study	NA	434	14–20	Not reported	Not reported	Reported that any contribution from supplements was not included in analysis			24 h recall (single)	Mean over pregnancy	−/+
Rycel et.al (2009) [44]	Poland	Retro-spective cohort	NA	102	15–18	Not reported	Not reported	none reported			Venous blood sample	Before and after delivery	−/+
Young et.al (2010) [29]	USA	Cross-sectional study	NA	92	14–18	Healthy, singleton pregnancy	HIV, diabetes, pre-eclampsia, eating disorders, malabsorption diseases, self-reported drug use	Prenatal supplement including 27 mg iron	92	Not reported	Venous blood sample	Second trimester and delivery	+
Young et.al (2012) [30]	USA	Cross-sectional study	NA	171	Under 18 (mean 17.1)	Healthy, singleton pregnancy	HIV, diabetes, pre-eclampsia, eating disorders, malabsorption diseases	400 IU Vitamin D supplement given to participants found to be deficient	46 (estimated from reported percentages)	26.4 % - daily, 35.8 % at least twice per week	10 ml venous blood sample	Delivery	+

Of the included studies, six provided information on dietary intakes only, 12 on biological markers only and three reported both types of information. Nutrient intakes from food sources were reported (therefore excluding any contribution from supplements) in all but one paper [23]. However, the majority (10 out of 15) of papers reporting biological markers also reported that participants were taking nutritional supplements, details of which along with other characteristics of the included studies are shown in Table 2. Due to inconsistencies in the type, dose, duration and compliance with supplement use it was not possible to quantify the impact of supplements on the results.

Of the 21 included studies 14 were carried out in the USA [24–37], one in the US territory of Guam [38], three in the UK [23, 39, 40] and one in each of Australia [41], Canada [42], Chile [43] and Poland [44]. Nutritional status was a primary outcome measure in all but one of the included studies where the primary outcomes were birth weight and prematurity [43].

The study designs of the included studies are listed within Table 2. The majority of the studies were cross-sectional surveys. Five studies were randomised controlled trials, where baseline dietary assessments before randomisation, or data from the control group only, permitted the inclusion of nutritional intake or biomarker data cross-sectionally. One study was a retrospective cohort analysis and one was a retrospective chart review.

Participants were all aged between 12 and 20 years with the majority being aged 16 and over. The majority of studies selected participants using convenience samples; other sampling methods used were purposive [26], representative probability sample [32], stratified random sample [44] and a retrospective medical chart review including all eligible records [37].

The majority of studies reported a range of ethnicities in the sample with the exception of three studies where all participants were African American [31, 36, 37], one including only Mexican American participants [28] and one where all participants were White [26]. Where reported the majority of participants had a BMI in the healthy range. Six studies reported participant’s weight gain from recruitment to delivery which ranged from 14 to 17 kg.

All but two of the studies reporting biological markers collected venous blood samples which were analysed in laboratories using standard testing procedures. One study which was a medical records review [37] did not provide details of how samples were collected. One study also assessed biological markers of calcium absorption using a 24 h urine collection followed by daily spot urine collections [33]. Data relating to participants nutrient intakes used a variety of data collection methods. Three studies used food diaries [24, 27, 39], one of which was weighed [27]. The remaining studies used single [28, 38, 41] or multiple [25, 43, 23] 24 h recalls. Four out of the nine studies reporting nutrient intakes stated that dietary assessments were carried out by a trained nutritionist or similar professional [25, 26, 43, 27].

### Nutrient intakes

Energy intake was reported by nine studies with seven of these reporting intakes below the recommendations at one or more time point (Table 3). Four studies also reported gestational weight gain which ranged from 14 to 17 kg. Pooled analysis of the percentage of the EAR for energy in these 10 studies revealed wide confidence limits around the estimated mean, with an average intake 9 % lower than the UK EAR (mean % EAR, 91.2 %, CI 29.6–152.8 %). Analysis of energy intake by trimester and study country of origin did not show any significant differences (UK studies 89.1 % CI 39.2–139.1 %, US studies 100.4 %, CI 24.2–176.6 %). Analysis of those studies reporting gestational weight gain only showed young women to be achieving a higher percentage of the EAR for energy (99.1 %, CI 41.0–157.2 %) than those studies which did not report weight gain (90.2 %, CI 27.9–152.5 %) but this difference was not statistically significant.

**Table 3:** Mean energy intake in individual studies compared to UK estimated average requirement

	Study	Second Trimester			Third Trimester			Mean over pregnancy		
		*N*=	Mean	Confidence Interval	*N*=	Mean	Confidence Interval	*N*=	Mean	Confidence Interval
Energy kcal/day ^a^	Baker (2009)				290	↓2147	2075, 2219			
	Castillo-Duran (2001) a	249	↓1887	1825, 1949	249	↓2030	1968, 2092			
	Castillo-Duran (2001) b	258	↓1863	1790, 1936	258	↓1982	1927, 2038			
	Chan (2006)	23	↔2223	2057, 2389	23	↓2276	2059, 2493			
	Derbyshire (2009)				20	↓2273	2052, 2494			
	Giddens (2000)							59	↓2342	2187, 2497
	Gutierrez (1999)	46	↔2390	2150, 2630	46	↔2620	2389, 2851			
	Job (1995)							70	↓2134	1949, 2320
	Lee (2013)				133	↓2303	2161, 2446	156	↔2273	2167, 2379
	Pobocik (2003)							434	↑2487	2388, 2586

^a^Comparison to UK EARs, First and Second Trimester requirement (Average UK EAR for Females aged 11–19) 2355 kcal/day, Third Trimester requirement 2546 kcal/day, Mean over pregnancy requirement (average of three trimester values) 2419 kcal/d, ↑ Study mean higher than reference value (*p* < 0.05), ↔ Study mean not different to reference value (*p* < 0.05), ↓ Study mean lower than reference value (*p* < 0.05)

Mean intakes of macronutrients are show in Table 4. Intakes of protein and total carbohydrate were roughly in line with recommendations. There were too few studies reporting intakes of total fat, fat types or sugars to permit conclusions to be drawn. Three studies reported any measurements of dietary fibre, all of which were below recommended levels.

**Table 4:** Mean intakes of macronutrients (g/day or percent of energy) and dietary fibre (g/day) in individual studies compared to UK and US dietary reference values

		Comparison to UK Dietary Reference Value									Comparison to US Dietary Reference Value								
Nutrient	Study	Second Trimester			Third Trimester			Mean over pregnancy			Second Trimester			Third Trimester			Mean over pregnancy		
		*N*=	Mean	Confidence Interval	*N*=	Mean	Confidence Interval	*N*=	Mean	Confidence Interval	*N*=	Mean	Confidence Interval	*N*=	Mean	Confidence Interval	*N*=	Mean	Confidence Interval
Protein grams/day ^a^	Castillo-Duran (2001) a	249	↑60	58, 62	249	↑62	59, 64				249	↓60	58, 62	249	↓62	59, 64			
	Castillo-Duran (2001) b	258	↑59	57, 61	258	↑60	58, 61				258	↓59	57, 61	258	↓60	58, 61			
	Chan (2006)	23	↑76	65, 87	23	↑76	65, 87				23	↔76	65, 87	23	↔76	65, 87			
	Derbyshire (2009)				20	↑72	65, 79							20	↔72	65, 79			
	Giddens (2000)							59	↑82	77, 87							59	↑82	77, 87
	Gutierrez (1999)	46	↑111	98, 124	46	↑118	105, 130				46	↑111	98, 124	46	↑118	105, 130			
	Job (1995)							70	↑73	73, 73							70	↑73	73, 73
	Lee (2013)				133	↑81	76, 87	156	↑70	65, 75				133	↑81	76, 87	156	↔70	65, 75
	Pobocik (2003)							434	↑99	95, 103							434	↑99	95, 103
Englyst Fibre grams/day ^b^	Derbyshire (2009)					↓12	11, 13												
AOAC Fibre grams/day ^c^	Giddens (2000)																59	↓14	13, 15
	Lee (2013)													133	↓13	12, 14	156	↓14	13,14
		*N*=	% of Energy		*N*=	% of Energy		*N*=	% of Energy										
Total Carbohydrate	Derbyshire (2009)				20	54 %													
	Giddens (2000)							59	50 %										
	Lee (2013)				133	51 %		156	52 %										
	Gutierrez (1999)	46	56 %		46	56 %													
Total Fat	Chan (2006)	23	29 %		23	29 %													
	Gutierrez (1999)	46	29 %		46	27 %													
Saturated Fat	Chan (2006)	23	9 %		23	10 %													
Total Sugars	Derbyshire (2009)				20	25 %													
Added Sugars	Lee (2013)				133	17 %		156	17 %										

^a^ Comparison to UK RNI 51 g/day and US RDA 71 g/day, ^b^ Comparison to UK RNI 18 g/day, ^c^ Comparison to US RDA 28 g/day, ↑ Study mean higher than reference value (*p* < 0.05), ↔ Study mean not different to reference value (*p* < 0.05), ↓ Study mean lower than reference value (*p* < 0.05)

Tables 5 and 6 show the pattern of micronutrient intakes across the included studies compared to UK and US Dietary Reference Values (DRVs).

**Table 5:** Intake of micronutrients in individual studies compared to UK and US dietary reference values - minerals

		Comparison to UK Dietary Reference Value									Comparison to US Dietary Reference Value								
Nutrient	Study	Second Trimester			Third Trimester			Mean over pregnancy			Second Trimester			Third Trimester			Mean over pregnancy		
		*N*=	Mean	Confidence Interval	*N*=	Mean	Confidence Interval	*N*=	Mean	Confidence Interval	*N*=	Mean	Confidence Interval	*N*=	Mean	Confidence Interval	*N*=	Mean	Confidence Interval
Calcium mg/day ^a^	Baker (2009)				290	↔840	800, 880							290	↓840	800, 880			
	Chan (2006)	23	↔835	711, 959	23	↔862	714, 1010				23	↓835	711, 959	24	↓862	714, 1010			
	Derbyshire (2009)				20	↑1007	867, 1147							20	↓1007	867, 1147			
	Giddens (2000)							59	↑989	904, 1074							59	↓989	904, 1074
	Gutierrez (1999)	46	↑1561	1334, 1789	46	↑1655	1424, 1886				46	↑1561	1334, 1789	46	↑1655	1424, 1886			
	Job (1995)							70	↔923	756, 1090							70	↓923	756, 1090
	Lee (2013)				133	↑916	838, 995	156	↑886	824, 948				133	↓916	838, 995	156	↓886	824, 948
	Pobocik (2003)							434	↓743	689, 797							434	↓743	689, 797
Phosphorous mg/day ^b^	Chan (2006)	23	↑934	811, 1057	23	↑961	812, 1110				23	↓934	811, 1057	24	↓961	812, 1110			
	Giddens (2000)							59	↑1340	1248, 1432							59	↔1340	1248, 1432
	Lee (2013)				133	↑1264	1182, 1347	156	↑1196	1131, 1261				133	↔1264	1182, 1347	156	↔1196	1131, 1261
	Pobocik (2003)							434	↑1338	1279, 1397							434	↑1338	1279, 1397
Iron mg/day ^c^	Baker (2009)				290	↑17	15, 19							290	↓17	15, 19			
	Castillo-Duran (2001) a	249	↑15.5	15, 16	249	↑16.8	16, 17				249	↓15.5	15, 16	249	↓16.8	16, 17			
	Castillo-Duran (2001) b	258	↑16.6	16, 17	258	↑16.8	16, 17				258	↓16.6	16, 17	258	↓16.8	16, 17			
	Chan (2006)	23	↑22	18, 26	23	↑25	20, 30				23	↓22	18, 26	23	↔25	20, 30			
	Derbyshire (2009)				20	↓12.6	11, 14							20	↔12.6	11, 14			
	Giddens (2000)							59	↔16	15, 17							59	↓16	15, 17
	Gutierrez (1999)	46	↑17.7	15, 20	46	↑22.7	17, 28				46	↓17.7	15, 20	46	↔22.7	17, 28			
	Job (1995)							70	↓11.2	10, 12							70	↓11.2	10, 12
	Lee (2013)				133	↑18.8	17, 20	156	↑18.6	17, 20				133	↓18.8	17, 20	156	↓18.6	17, 20
	Pobocik (2003)							434	↑20	19, 21							434	↓20	19, 21
Magnesium mg/day ^d^	Baker (2009)				290	↓236	227, 245								↓236	227, 245			
	Chan (2006)	23	↓263	230, 296	23	↓264	231, 297					↓263	230, 296		↓264	231, 297			
	Derbyshire (2009)				20	↓244	218, 270								↓244	218, 270			
	Giddens (2000)							59	↓252	234, 270								↓252	234, 270
	Lee (2013)				133	↓237	222, 253	156	↓231	218, 244					↓237	222, 253		↓231	218, 244
	Pobocik (2003)							434	↓270	258, 282								↓270	258, 282
Potassium mg/day ^e^	Chan (2006)	23	↓2802	2512, 3092	23	↓2954	2635, 3273				23	↓2802	2512, 3092	23	↓2954	2635, 3273			
	Derbyshire (2009)				20	↓2948	2659, 3237							20	↓2948	2659, 3237			
Zinc mg/day ^f^	Baker (2009)				290	↑8.1	7.8, 8.4							290	↓8.1	7.8, 8.4			
	Castillo-Duran (2001) a	249	↑7.4	7.1, 7.7	249	↑7.7	7.4, 8				249	↓7.4	7.1, 7.7	249	↓7.7	7.4, 8			
	Castillo-Duran (2001) b	258	↑7.4	7.1, 7.7	258	↑7.4	7.2, 7.6				258	↓7.4	7.1, 7.7	258	↓7.4	7.2, 7.6			
	Chan (2006)	23	↑16	11.9, 20.1	23	↑18	13.5, 22.5				23	↔16	11.9, 20.1	23	↑18	13.5, 22.5			
	Derbyshire (2009)				20	↑8.1	7.4, 8.9							20		7.4, 8.9			
	Giddens (2000)							59	↑11.6	10.5, 12.7							59	↑11.6	10.5, 12.7
	Gutierrez (1999)	46	↑14.5	12.6, 16.4	46	↑15.3	13.4, 17.2				46	↑14.5	12.6, 16.4	46	↑15.3	13.4, 17.2			
	Job (1995)							70	↑9.5	8.5, 10.5							70	↑9.5	8.5, 10.5
	Lee (2013)				133	↑12.8		156	↑12.6	11.4, 13.8				133	↑12.8		156	↑12.6	11.4, 13.8
	Pobocik (2003)							434	↑13	12.2, 13.8							434	↑13	12.2, 13.8
Sodium mg/day ^g^	Chan (2006)	23	↑3316	2809, 3823	23	↑3323	2812, 3834					↑3316	2809, 3823		↑3323	2812, 3834			
	Derbyshire (2009)				20	↑3089	2722, 3456								↑3089	2722, 3456			
Copper μg/day ^h^	Giddens (2000)							59	↑1200	1098, 1302							59	↑1200	1098, 1302
	Lee (2013)				133	↑1100	1015, 1185	156	↑1085	1021, 1151				133	↑1100	1015, 1185	156	↑1085	1021, 1151
Selenium μg/day ^i^	Giddens (2000)							59	↑116	109, 123							59	↑116	109, 123

^a^Comparison to UK RNI 800 mg/day and US RDA 1300 mg/day, ^b^Comparison to UK RNI 625 mg/day and US RDA 1250 mg/day, ^c^Comparison to UK RNI 14.8 mg/day and US RDA 27 mg/day, ^d^Comparison to UK RNI 300 mg/day and US RDA 400 mg/day, ^e^Comparison to UK RNI 3500 mg/day and US RDA 4700 mg/day, ^f^Comparison to UK RNI 7 mg/day and US RDA 12 mg/day, ^g^Comparison to UK RNI 1500 mg/day and US RDA 1600 mg/day, ^h^Comparison to UK RNI 1000 μg/day and US RDA 1000 μg/day, ^i^Comparison to UK RNI 60 μg/day and US RDA 60 μg/day, ↑ Study mean higher than reference value (*p* < 0.05), ↔ Study mean not different to reference value (*p* < 0.05), ↓ Study mean lower than reference value (*p* < 0.05)

**Table 6:** Intake of micronutrients in individual studies compared to UK and US dietary reference values - vitamins

		Comparison to UK Dietary Reference Value									Comparison to US Dietary Reference Value								
Nutrient	Study	Second Trimester			Third Trimester			Mean over pregnancy			Second Trimester			Third Trimester			Mean over pregnancy		
		*N*=	Mean	Confidence Interval	*N*=	Mean	Confidence Interval	*N*=	Mean	Confidence Interval	*N*=	Mean	Confidence Interval	*N*=	Mean	Confidence Interval	*N*=	Mean	Confidence Interval
Vitamin D μg/day ^a^	Baker (2009)				290	↓2.1	2, 2.3							290	↓2.1	2, 2.3			
	Chan (2006)	23	↓2.8	1.9, 3.7	23	↓3.1	2.2, 4				23	↓2.8	1.9, 3.7	23	↓3.1	2.2, 4			
	Derbyshire (2009)				20	↓2.0	1.5, 2.6							20	↓2.0	1.5, 2.6			
	Giddens (2000)							59	↓6.4	5.7, 7.1							59	↓6.4	5.7, 7.1
	Lee (2013)				133	↓5.4	4.7, 6.1	156	↓5.1	4.6, 5.6				133	↓5.4	4.7, 6.1	156	↓5.1	4.6, 5.6
Vitamin E ^b^	Baker (2009)													290	↓8.9	8.3, 9.5			
	Derbyshire (2009)													20	↓7.7	6.5, 8.9			
	Gutierrez (1999)										46	↓10.7	10.7	46	↓11.2	8.2, 14.1			
	Lee (2013)													133	↓6.9	6.2, 7.6	156	↓6.9	6.4, 7.4
	Pobocik (2003)																434	↓8	7.2, 8.8
Vitamin C ^c^	Baker (2009)				290	↑160	146, 174							290	↑160	146, 174			
	Derbyshire (2009)				20	↑138	111, 165							20	↑138	111, 165			
	Giddens (2000)							59	↑128	112, 144							59	↑128	112, 144
	Gutierrez (1999)	46	↑252	208, 296	46	↑231	190, 271				46	↑252	208, 296	46	↑231	190, 271			
	Job (1995)							70	↑135	92, 178							70	↑135	92, 178
	Lee (2013)				133	↑97	81, 113	156	↑106	94, 118				133	↑97	81, 113	156	↑106	94, 118
	Pobocik (2003)							434	↑167	150, 184							434	↑167	150, 184
Folate ^d^	Baker (2009)				290	↔285	269, 301							290	↓285	269, 301			
	Derbyshire (2009)				20	↓227	205, 249							20	↓227	205, 249			
	Giddens (2000)							59	↔312	277, 347							59	↓312	277, 347
	Gutierrez (1999)	46	↑447	355, 540	46	↑393	340, 445				46	↓447	355, 540	46	↓393	340, 445			
	Lee (2013)				133	↑829	723, 935	156	↑849	645, 1053				133	↑829	723, 935	156	↑849	645, 1053
	Pobocik (2003)							434	↔292	269, 315							434	↓292	269, 315
Riboflavin ^e^	Chan (2006)	23	↑2.3	1.8, 2.8	23	↑2.4	2, 2.8				23	↑2.3	1.8, 2.8	23	↑2.4	2, 2.8			
	Giddens (2000)							59	↑2.3								59	↑2.3	2.1, 2.5
	Lee (2013)				133	↑2.5	2.3, 2.7	156	↑2.4					133	↑2.5	2.3, 2.7	156	↑2.4	2.2, 2.6
	Pobocik (2003)							434	↑2.1								434	↑2.1	2, 2.2
B12 ^f^	Baker (2009)				290	↑5.3	4.7, 5.9							290	↑5.3	4.7, 5.9			
	Chan (2006)	23	↑5	3.8, 6.2	23	↑5.2	3.7, 6.7				23	↑5	3.8, 6.2	23	↑5.2	3.7, 6.7			
	Giddens (2000)							59	↑5.3	4.6, 6							59	↑5.3	4.6, 6
	Lee (2013)				133	↑5.6	5, 6.2	156	↑5.5	5, 6				133	↑5.6	5, 6.2	156	↑5.5	5, 6
	Pobocik (2003)							434	↑5.5	4.8, 6.2							434	↑5.5	4.8, 6.2
Thiamin ^g^	Baker (2009)				290	↑1.6	1.5, 1.7							290	↑1.6	1.5, 1.7			
	Giddens (2000)							59	↑2.1	1.9, 2.3							59	↑2.1	1.9, 2.3
	Lee (2013)				133	↑2.1	1.9, 2.3	156	↑2.1	1.9, 2.3				133	↑2.1	1.9, 2.3	156	↑2.1	1.9, 2.3
	Pobocik (2003)							434	2.4	2.3, 2.5							434	2.4	2.3, 2.5
Niacin ^h^	Baker (2009)				290	↑33	32, 35							290	↑34	32, 35			
	Giddens (2000)							59	↑24	22, 26							59	↑24	22, 26
	Lee (2013)				133	↑28	26, 30	156	↑26	24, 27				133	↑28	26, 30	156	↑26	24, 27
	Pobocik (2003)							434	↑30	29, 31							434	↑30	29, 31
B6 ^i^	Baker (2009)				290	↑2.3	2.1, 2.4							290	↑2.3	2.1, 2.4			
	Giddens (2000)							59	↑1.9	1.7, 2.1							59	↑1.9	1.7, 2.1
	Lee (2013)				133	↑2.2	2, 2.4	156	↑2.1	1.9, 2.3				133	↑2.2	2, 2.4	156	↑2.1	1.9, 2.3
	Pobocik (2003)							434	↑2	1.9, 2.1							434	↑2	1.9, 2.1
Vitamin A ^j^	Baker (2009)				290	↑759	651, 867							290	↑759	651, 867			
	Derbyshire (2009)				20	↑555	439, 671							20	↓555	439, 671			
	Giddens (2000)					↑105		59	↑1053	907, 1199					↑1053		59	↑1053	907, 1199
	Gutierrez (1999)	46	↑2492	1466, 3518	46	↑197	1432, 2523				46	↑2492	1466, 3518	46	↑1978	1432, 2523			
	Job (1995)							70	↑973	802, 1144							70	↑973	802, 1144
	Lee (2013)				133	↑698	614, 783	156	↑666	615, 717				133	↑698	614, 783	156	↓666	615, 717
	Pobocik (2003)							434	↑109	944, 1242							434	↑1093	944, 1242
Vitamin K ^k^	Lee (2013)													133	↔70	57, 83	156	↔70	59, 81

^a^ Comparison to UK RNI 10 μg/day and US RDA 15 μg/day, ^b^ Comparison to US RDA 15 mg/day, ^c^ Comparison to UK RNI 40 mg/day and US RDA 80 mg/day, ^d^ Comparison to UK RNI 200 μg/day and US RDA 600 μg/day, ^e^ Comparison to UK RNI 1.1 mg/day and US RDA 1.4 mg/day, ^f^ Comparison to UK RNI 1.5 μg/day and US RDA 2.6 μg/day, ^g^ Comparison to UK RNI 0.8 mg/day and US RDA 1.4 mg/day, ^h^ Comparison to UK RNI 14 mg/day and US RDA 18 mg/day, ^i^ Comparison to UK RNI 1.2 mg/day and US RDA 1.9 mg/day, ^j^ Comparison to UK RNI 600 μg/day and US RDA 750 μg/day, ^k^ Comparison to US AI 75 μg/day, ↑ Study mean higher than reference value (*p* < 0.05), ↔ Study mean not different to reference value (*p* < 0.05), ↓ Study mean lower than reference value (*p* < 0.05)

The individual study results show that the majority of reported nutrient intakes were significantly below both the UK RNI and US RDA for vitamin D, potassium and magnesium and below the US RDA for calcium, vitamin E, folate, phosphorous and iron. Zinc intakes reported as the mean intake over pregnancy were low whereas this was not the case in the studies reporting intakes in the second or third trimesters specifically.

**Fig. 2:**
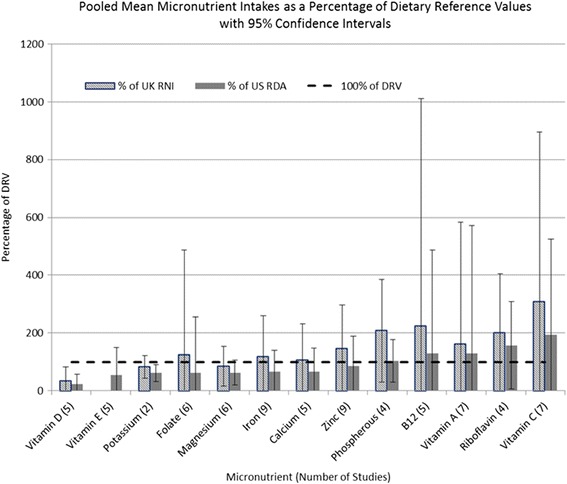
Pooled weighted mean micronutrient intake compared to UK and US dietary reference values

Results of the pooled analyses however showed that only intake of vitamin D remained significantly below both the UK RNI and US RDA, and intakes of potassium below the US RDA. Sub-group analysis showed that micronutrient intake was lower in UK based studies than those based in the USA for all micronutrients with the exception of vitamin C, however vitamin D was the only micronutrient where the percentage of the DRV in UK based studies was below the UK RNI (21.4 %, CI 0–63.5 %) and US RDA (14.3 %, CI 0–42.3 %). Results of the pooled analysis of micronutrients are shown in Fig. 2. Detailed results of the sub-group analysis of nutrient intakes are available in Additional file 1: Table S2 and Additional file 1: Table S3.

Micronutrient analysis by stage of pregnancy showed that intake of vitamin D in the second (28 % of the UK RNI, CI 26–30 %) and third (31.2 % of the UK RNI, CI 0–47 %) trimesters was below recommendations; this was not the case for measures reported as a mean over pregnancy (54 % of the UK RNI, CI 0–114.3 %). In the third trimester the percentage of the US RDA represented by intakes of magnesium (19.9 %, CI 20.4–98.6 %) and potassium (62.8 %, CI 32.5–93.1 %) were also below recommendations.

### Biological markers

Table 7 shows the pattern of biological markers reported across the included studies compared to WHO, UK and US minimum thresholds for deficiency where available. As the recommended cut off points given by all three authorities are consistent, the results are presented in one combined table for clarity. Other nutrients were considered compared to deficiency thresholds suggested in the academic literature as previously discussed.

**Table 7:** Biological markers of nutritional status in individual studies compared to reference values

	Study ID	First Trimester			Second Trimester			Third Trimester			Delivery		
		*N*=	Mean	Confidence Interval	*N*=	Mean	Confidence Interval	*N*=	Mean	Confidence Interval	*N*=	Mean	Confidence Interval
Haemoglobin (g/L) ^a^	Baker (2009)	404	↑122	121, 123				362	↓108	107, 109			
	Chang (2003)	445	↑121	121, 122	319	↑112	112, 114	836	↓107	107, 109			
	Dawson (2000) a				21	↑124	123, 125	21	↑115	114, 116	20	↑114	111, 112
	Dawson (2000) b				20	↑125	124, 126	20	↑119	118, 120	21	↑116	112, 114
	Dawson (2000) a							21	↑114	113, 115			
	Dawson (2000) b							20	↑120	119, 121			
	Gadowsky (1995)							50	↑119	115, 123			
	Iannotti (2005)				35	↔111	107, 115	70	↔107	104, 110			
	McGuire Davies (2010)				78	↑118	118, 118	76	↓107	107, 108			
	Meier (2002) a				20	↑125	122, 129	15	↑123	119, 128			
	Meier (2002) b				17	↑128	124, 132	16	↑117	112, 123			
	Meier (2002) a				19	↑116	112, 120						
	Meier (2002) b				16	↑113	110, 116						
	Rycel (2009)										102	↑120	120, 229
	Rycel (2009)										102	↓103	103, 103
	Young (2010)				48	↑113					62	↑117	113, 121
Ferritin (μg/L or ng/ml) ^b^	Gadowsky (1995)				50	↓7.4	5.7, 9.1						
	Iannotti (2005)				44	↑33	26.8, 40.6	59	↔15	12.6, 17.8			
	Meier (2002) a				19	↑42	31.3, 52.8	15	↔25	14.6, 35.4			
	Meier (2002) b				17	↑57	36.5, 77.5	15	↓6.8	5.2, 8.5			
	Meier (2002) a				19	↑46	24, 68.8						
	Meier (2002) b				16	↓10	7.9, 13						
	Young (2010)				81	↔16	12.9, 20.1				88	↔17	14.9, 20.3
Haematocrit ^(g/L) c^	Chang (2003)	445	↑36	35.7, 36.3	319	↔33	32.7, 33.3	836	↓32	31.8, 32.2			
	Gadowsky (1995)							50	↑36	34.8, 37.2			
	Iannotti (2005)				35	↔33	32, 34	70	↓32	31.3, 32.7			
	Rycel (2009)										102	34	34.6, 34.8
	Rycel (2009)										102	↓31	31.4, 31.6
Zinc μmol/L-1 ^d^	Castillo-Duran (2001) a	249	↑11.9	11.7, 12.1				249	↑10.9	10.7, 11.1			
	Castillo-Duran (2001) b	258	↑11.7	11.5, 11.9				258	↑10.9	10.7, 11.1			
	Castillo-Duran (2001) a							249	↑10.5	10.3, 10.7			
	Castillo-Duran (2001) b							258	↑10.2	10, 10.4			
	Chan (2006)										23	↑26.3	23.2, 29.4
	Mistry (2014) a							107	↑9.71	8.8, 10.5			
	Mistry (2014) b							19	↑10.8	7.8, 13.9			
Magnesium mmol/l ^e^	Chan (2006)										23	↑0.99	0.9, 1.1
Phosphorous mg/dl ^f^	Chan (2006)										23	↑5	4, 6
Copper μg/dl ^g^	Mistry (2014) a							107	↑206	1991, 2128			
	Mistry (2014) b							19	↑196	1712, 2207			
Selenium μg/L ^h^	Dawson (2000) a				21	↓49	46, 52	21	↓55	52.9, 57.1	21	↑114	108.9, 119.1
	Dawson (2000) b				20	↓44	39.6, 48.4	20	↓53	50.8, 55.2	20	↓55	50.2, 59.8
	Dawson (2000) a				21	↓50	46.2, 53.8	21	↔85	76.9, 93.1			
	Dawson (2000) b				20	↓44	39.6, 48.4	20	↓62	57.2, 66.8			
	Mistry (2014) a							107	↓65	62.7, 67.5			
	Mistry (2014) b							19	↓49	45.9, 52.9			
Red blood cell folate (nmol/l) ^i^	Baker (2009)							266	↑647	616, 680			
Serum folate (nmol/l) ^j^	Baker (2009)							291	↑12	12, 14			
	Chan (2006)										23	↔13	10, 17
Vitamin A (μg/dL)	Chan (2006)										23	↑38	31, 45
B12 (pmol/l) ^k^	Baker (2009)							293	↑177	169, 185			
	Chan (2006)										23	↑265	216, 315
	Gadowsky (1995)							50	↑170	146, 194			
Homocysteine (μmol/L) ^l^	Baker (2009)							293	7.9	7.6, 8.2			
	Gadowsky (1995)							50	6.1	3, 9			
25OHD (nmol/L) ^m^	Baker (2009)							263	↑33	30.4, 35.8			
	Chan (2006)										23	↑57	48, 67
	Ginde (2010)												
	McGuire Davies (2010)				44	↑52	46, 58	36	↑56	50, 63			
	O’Brien (2003)							23	↑50	41, 60			
	Young (2012)										171	↑54	51, 59

^a^ Target value 110 g/L, ^b^ Target value 15 μg/L, ^c^ Target value33 g/L, ^d^ Target value 6.12 μmol/L-1, ^e^ Target value 0.9 mmol/L, ^f^ Target value 2.5 mg/dl, ^g^ Target value 63.7 μg/dl, ^h^ Target value 90 μg/L, ^i^ Target value 340 nmol/L, ^j^ Target value 20 μg/dl, ^k^ Target value 150pmol/L, ^l^ Target value less than 13 μmol/L, ^m^ Target value 25 nmol/L, ↑ Study mean higher than reference value (*p* < 0.05), ↔ Study mean not different to reference value (*p* < 0.05), ↓ Study mean lower than reference value (*p* < 0.05)

The results show that the mean reported biomarker values in the majority of studies suggested that young women’s nutritional status was sufficient, with the exception of markers of iron and selenium status. Results for haematocrit and plasma ferritin were mixed, with results being more likely to be below the cut off in the third trimester and at delivery. Measures of serum selenium were reported to be less than the cut off in the majority of studies.

Examination of pooled, weighted means showed that only mean selenium concentration was below the reference value. The weighted means for all biological markers where there were two or more valid results are shown in Table 8.

**Table 8:** Pooled weighted means of biological markers

Biological Marker	Pooled Mean	Confidence Interval	Target Value	Number of studies
Haemoglobin (g/dL)	112.4	95.4–133.8	110	9
25_OH_D (nmol/L)	55.9	6.2–105.7	25	6
Ferritin (μg/L)	18.2	0–48.3	15	4
Haematocrit (g/L)	32.5	28.0–39.4	33	4
Zinc (μmol/L-1)	11.5	5.9–17.1	6.12	3
B12 (pmol/L)	181.6	0–271.1	150	3
Serum Folate (nmol/L)	12.8	0–26.8	10	2
Selenium (μg/L)	61.8*	39.2–84.4	90	2
Homocystine (μmol/L)	7.6	0–17.0	Less than 13	2

* Significant at the *p*<0.005 level

The sub-group analysis by country of origin was only possible by US vs. non-US studies as there was only one UK based study reporting biological markers. The analysis failed to detect any differences by study country of origin. Analysis by stage of pregnancy suggests a decline in iron status markers haemoglobin, haematocrit and ferritin as pregnancy progresses; the levels observed however do not necessarily reflect iron deficiency. Detailed results of the sub-group analysis of biological markers are available in Additional file 1: Table S4 and Additional file 1: Table S5.

## Discussion

Compared with reviews of the nutritional status of pregnant adolescents published in 2007, this review identified a further 13 studies that reported data on nutritional intakes and biomarkers of status. The summary results show that there may be areas of concern in adolescent’s nutritional intake during pregnancy, particularly compared to US recommendations, with regard to calcium, vitamin D, vitamin E, folate, potassium and magnesium. The evidence also suggests that overall energy intake may be lower than recommended.

In terms of comparison with dietary reference values, combined analysis of the individual study means showed very few statistically significant results, with the exception of vitamin D. One possible explanation for this is that there was a high level of variance between participants in the majority of studies resulting in very wide confidence intervals. This suggests that there may be sub-groups of young women within the total population who are at higher risk of poor nutritional status which this review has failed to detect. Differences in micronutrient intake were observed between UK and USA based studies which may in part be explained by routine fortification of food products in the USA compared to the UK. Only one study reported that supplements were included in the reported intake values, and the compliance rates for supplements in this study were low, meaning that the impact of supplement use on intakes data is marginal.

Macronutrient contributions to energy were found to be roughly in line with recommendations, however there was a significant lack of data for carbohydrates (including sugars and fibre) and fats (including saturated fat) meaning these results should be interpreted with caution. Further research into macronutrient intakes in this population, particularly with regard to types of carbohydrates and fats, is needed.

The methods used to assess dietary intake varied across the included studies. The two methods reported by the included studies were 24 h dietary recalls (single and multiple) and food diaries. While these are validated and accepted methods of nutritional surveillance [45], it is acknowledged that underreporting biases may exist [46] and so results should be considered with this in mind.

Four of the studies reporting energy intake also reported mean gestational weight gain which ranged from 14 kg to 17 kg, consistently higher than the required pregnancy weight gain [47]. Mean percentage intake of the EAR for energy was higher in the studies reporting weight gain than those which did not report this measure, but not significantly so. This is potentially contradictory of the finding that energy intake was low in individual studies and suggests further work is needed regarding the potential level of under reporting in this population and the relationship between dietary patterns, overall energy consumption and gestational weight gain.

Inadequacies in nutrient intakes did not necessarily translate to systemic deficiencies as measured by mean values of biological markers, with the possible exceptions of markers of iron and selenium status. One possible explanation for this is that food intake may have been under reported therefore suggesting that intake was insufficient when this was not the case. A further possible explanation is that measures of biological markers were elevated by dietary supplements. Details regarding the type, dose, duration and number of participants taking supplements were inconsistent in the included papers meaning that detailed analysis of the impact of supplement use on nutritional status was not possible, however 10 out of the 15 included studies reporting biomarkers did report some level of supplementation. This finding does suggest that supplements may play an important role in ensuring young women do not experience nutrient insufficiency, however attention to clear reporting of supplement use in research papers is essential to allow a better understanding of the impact of supplementation on nutritional status.

The participants in all of the included studies where supplements were provided may also have been more compliant with taking supplements due to the very fact that they were taking part in a research study than might be expected outside of a study environment. A systematic review [48] of the effect of dietary interventions in adolescent pregnancies found some evidence to suggest that nutritional supplements may reduce the likelihood of low birth weight; however the review also reported a serious lack of good quality research papers in this area. Further work to establish the extent to which pregnant young women in the general population suffer more from nutrient deficiencies and the impact of supplement use would be advantageous.

There is significant evidence in the literature regarding the role of nutrition in supporting healthy pregnancies and allowing the foetus to achieve its full potential. Adolescent girls are at particular risk of iron deficiency anaemia due in part to rapid growth during adolescence [49] combined with the onset of menarche. This coupled with the increased demand for iron in pregnancy for expansion of maternal tissues and foetal growth, makes pregnant adolescents a particularly vulnerable group. There is some evidence to suggest that iron deficiency is implicated in the risk of adverse birth outcomes such as prematurity and low birth weight [50], meaning this is potentially an important factor in improving maternal and infant health. While analysis of mean values for markers of iron deficiency in this review did not indicate a significant issue, consideration of the reported prevalence of iron deficiency anaemia in the included studies suggests that this may be a concern for this population.

Vitamin D and calcium have an essential role in the mineralization of the developing foetal skeleton and insufficient intake of these nutrients may impact on foetal bone growth. The interaction between these two nutrients has been shown to be key to maximising foetal bone growth in pregnant adolescents and that growth is adversely affected when either of the two nutrients were lacking [30]. The pooled mean for vitamin D status as reflected in blood 25 (OH)D in this review was significantly below recommended levels across all trimesters. It was not possible to conduct analysis of vitamin D status by ethnicity or exposure to sunlight, however, which are factors known to have a significant impact on vitamin D status [51–53].

The role of folate in the prevention of neural tube defects in early pregnancy has been well documented [54]. There is a lack of data collected in early pregnancy in the papers included in the current review, however the observed failure to meet recommendations for folate intake in later pregnancy reported may suggest that the participants were unlikely to have been meeting recommendations prior to taking part in a research study. A systematic review of the impact of folate intake over the course of pregnancy [55] also found a significant effect in the second and third trimesters on infant birth weight, suggesting that the importance of folate for a healthy pregnancy extends beyond the first trimester.

Selenium status has been identified as a potential area of concern in this review. Selenium is a trace element which has an anti-oxidative effect and protects cell membranes [56]. The target value used to assess selenium status was based on the intake necessary for maximisation of plasma glutathione peroxidase activity, which is the criteria used in the derivation of the US RDA [18]. Selenium status has been shown to be associated with a number of adverse outcomes for both mother and child including neural tube defects [57], lower birth weights [58], cholestasis [59] and gestational diabetes [60].

While demographic characteristics of participants were reported in the included studies data was generally reported for the study population as a whole, meaning that sub-group analysis was not possible. This is significant in that evidence suggests that younger adolescents, those who smoke and those from more deprived backgrounds may be at higher risk of nutritional issues [3, 61, 62].

There is also evidence to suggest that the nutritional status of adult pregnant women may raise similar concerns to those identified within this review. A systematic review of micronutrient intakes in pregnancy found that intakes of folate, vitamin D and iron were sub-optimal [63]. A further review focusing on energy and macronutrient intake in this population found that intakes of energy and fibre were also below recommendations [64]. These results are consistent with the findings of this review suggesting that maternal age alone may not be the most important factor in sub-optimal nutritional status during pregnancy. Further work to identify the characteristics of those most at risk, particularly within the adolescent population, and the nature of that risk is needed.

### Limitations

There were some significant limitations which impact on the conclusions of this review. The majority of the included papers used convenience samples meaning that there is likely to be an element of bias in the reported outcomes. The majority of participants in the included studies were aged 16 and over meaning that the results may not be generalisable to younger adolescents, who may also be at greater nutritional risk compared to older adolescents due to competing growth needs [3]. The lack of detail regarding participant’s supplement use meant that it was not possible to evaluate the impact of supplements on biological markers of nutritional status. It is therefore likely that these results may have been biased by supplement use in some participants.

There was significant heterogeneity in the included papers in terms of study design. Measurements of dietary intake differed between papers however the majority of studies used 24 h recall methods to assess nutrient intake. This has been shown to have limitations in terms of both participants reporting their intake accurately and the likelihood that the recorded intake is representative of the usual diet, particularly in adolescents [46]. Three studies used multiple 24 h recalls in order to produce more reliable estimates of intakes; however this approach was not consistent across the included studies using this method.

There were also considerable differences in the number of nutritional indicators represented. Pooled means were calculated wherever two or more data points were available in order to maximise the results available from the review. This however means that some estimates will be more robust than others depending on the number of data points on which they are based. There was a large degree of variation in the amount of data available for different nutrients, for example assessment of serum selenium was based on data from only two papers whereas nine independent studies contributed data on haemoglobin concentration.

The pooling of study means gives a useful indication of potential inadequacies across the population as a whole; however this approach lacks the sensitivity to draw conclusions regarding the prevalence of nutrient deficiencies in the population. Examination of the reported prevalence of deficiency in the included studies shows results which are inconsistent with the analysis based on study means. The prevalence of iron deficiency anaemia measured by haemoglobin concentration reportedly ranged from 1.2 to 63.5 %, with prevalence in the third trimester ranging from 29 to 63.5 %. Other markers of iron status followed a similar pattern with higher prevalence of deficiency occurring in the third trimester. The prevalence of vitamin D deficiency in many of these studies was also higher than suggested by the analysis of study means. This suggests, similarly to the data regarding nutrient intakes, that there are substantial skews within the data and sub-groups of young women who may be more at risk of deficiencies.

## Conclusion

This review identifies some concerns in the nutritional status of pregnant adolescents which may impact on maternal and infant outcomes. Intake of vitamin D and serum selenium status were identified as being significantly low in pooled analysis of included studies. Fibre intake was also below recommendations.. This said there are some significant limitations meaning these results should be interpreted with caution. No analysis of the effect of demographic characteristics on either nutritional intake or biological markers was possible, nor was it possible to examine the impact of supplement use on biological markers.

Patterns in this population are also similar to those reported in the adult population during pregnancy. These findings suggest that targeted work to identify those most at risk, and the nature of that risk, is needed. Recommendations for other areas of further research include the macronutrient composition of adolescent’s diet during pregnancy, the relationship between nutrient intakes and birth outcomes and the role of nutritional supplements in this population.

## Additional file

Additional file 1:
**Table S1.** Characteristics of Excluded Studies. **Table S2.** Pooled, Weighted Mean Nutrient Intakes Expressed as Percentages of Dietary Reference Values by Stage of Pregnancy. **Table S3.** Pooled, Weighted Mean Nutrient Intakes Expressed as Percentages of Dietary Reference Values by Study Country of Origin. **Table S4.** Pooled, Weighted Mean Biological Markers of Nutritional Status by Stage of Pregnancy. **Table S5.** Pooled, Weighted Mean Biological Markers of Nutritional Status by Study Country of Origin. (DOCX 57 kb)

## Abbreviations

CASP: Critical appraisal and skills program
DRV: Dietary reference value
EAR: Estimated average requirement
RDA: Recommended daily allowance
RNI: Reference nutrient intake
WHO: World Health Organisation
