# Supplementary material for: Nutrient intakes and nutritional biomarkers in pregnant adolescents: a systematic review of studies in developed countries
Source: BMC Pregnancy Childbirth. 2016 Sep 15;16:268. doi: 10.1186/s12884-016-1059-9 (PMC5024513; doi:10.1186/s12884-016-1059-9)
Supplement: Additional file 1: — Table S1. Characteristics of Excluded Studies. Table S2. Pooled, Weighted Mean Nutrient Intakes Expressed as Percentages of Dietary Reference Values by Stage of Pregnancy. Table S3. Pooled, Weighted Mean Nutrient Intakes Expressed as Percentages of Dietary Reference Values by Study Country of Origin. Table S4. Pooled, Weighted Mean Biological Markers of Nutritional Status by Stage of Pregnancy. Table S5. Pooled, Weighted Mean Biological Markers of Nutritional Status by Study Country of Origin. (DOCX 57 kb) [file 12884_2016_1059_MOESM1_ESM.docx]

Additional file 1: Table S1. Characteristics of Excluded Studies

| Reference | Reason for exclusion |
| --- | --- |
| Briggs, M. M., Hopman, W. M., & Jamieson, M. A. (2007). Comparing pregnancy in adolescents and adults: obstetric outcomes and prevalence of anemia. *Journal of obstetrics and gynaecology Canada: JOGC= Journal d'obstetrique et gynecologie du Canada: JOGC*, *29*(7), 546-555. | Anaemia is the only relevant outcome measure and is only reported as a binary measure |
| O'Brien, K. O., Donangelo, C. M., Ritchie, L. D., Gildengorin, G., Abrams, S., & King, J. C. (2012). Serum 1, 25-dihydroxyvitamin D and calcium intake affect rates of bone calcium deposition during pregnancy and the early postpartum period. *The American journal of clinical nutrition*, *96*(1), 64-72. | No baseline nutritional measures and no control group intakes |
| De Vienne, C. M., Creveuil, C., & Dreyfus, M. (2009). Does young maternal age increase the risk of adverse obstetric, fetal and neonatal outcomes: a cohort study. *European Journal of Obstetrics & Gynecology and Reproductive Biology*, *147*(2), 151-156. | Anaemia is the only relevant outcome measure and is only reported as a binary measure |
| Dean, S. V., Lassi, Z. S., Imam, A. M., & Bhutta, Z. A. (2014). Preconception care: nutritional risks and interventions. *Reprod Health*, *11*(Suppl 3), S3. | Review - considers preconception nutritional interventions |
| Garikapaty, V. P., Feyerharm, R., & Zhu, B. (2007). Folic acid consumption among Missouri women in the periconceptional period. *Missouri medicine*, *105*(6), 504-509. | Peri-conceptional period only |
| Gupta, N., Kiran, U., & Bhal, K. (2008). Teenage Pregnancies: Obstetric characteristics and outcome. *European Journal of Obstetrics & Gynecology and Reproductive Biology*, *137*(2), 165-171. | Anaemia is the only relevant outcome measure and is only reported as a binary measure |
| Jones, R. L., Cederberg, H. M. S., Wheeler, S. J., Poston, L., Hutchinson, C. J., Seed, P. T., ... & Baker, P. N. (2010). Relationship between maternal growth, infant birthweight and nutrient partitioning in teenage pregnancies. *BJOG: An International Journal of Obstetrics & Gynaecology*, *117*(2), 200-211. | Data is split into 'growers' and 'non-growers', same data as Baker 2009 which is included |
| Oliveira, O. R., Santana, M. G., Santos, F. S., Conceição, F. D., Sardinha, F. L., Veiga, G. V., & do Carmo, M. G. T. (2012). Composition of fatty acids in the maternal and umbilical cord plasma of adolescent and adult mothers: relationship with anthropometric parameters of newborn. *Lipids in health and disease*, *11*(1), 1. | Country of origin excluded - Brazil |
| Tsikouras, P., Dafopoulos, A., Trypsianis, G., Vrachnis, N., Bouchlariotou, S., Liatsikos, S. A., ... & Von Tempelhoff, G. F. (2012). Pregnancies and their obstetric outcome in two selected age groups of teenage women in Greece. *The Journal of Maternal-Fetal & Neonatal Medicine*, *25*(9), 1606-1611. | Anaemia is the only relevant outcome measure and is only reported as a binary measure |
| Wheeler, S. J., Poston, L., Thomas, J. E., Seed, P. T., Baker, P. N., & Sanders, T. A. (2011). Maternal plasma fatty acid composition and pregnancy outcome in adolescents. *British journal of nutrition*, *105*(04), 601-610. | Oily fish is the only intake variable reported, data is from the same study as Baker 2009 which is included |
| Wise, N. J., & Arcamone, A. A. (2011). Survey of adolescent views of healthy eating during pregnancy. *MCN: The American Journal of Maternal/Child Nursing*, *36*(6), 381-386. | Does not report a valid comparable measure of dietary intake or nutritional status |
| Young, B. E., McNanley, T. J., Cooper, E. M., McIntyre, A. W., Witter, F., Harris, Z. L., & O'Brien, K. O. (2012). Vitamin D insufficiency is prevalent and vitamin D is inversely associated with parathyroid hormone and calcitriol in pregnant adolescents. *Journal of Bone and Mineral Research*, *27*(1), 177-186. | Focus on effect of hormone changes, reports same data Young 2012 which is included |
| Finkelstein, J. L., Pressman, E. K., Cooper, E. M., Kent, T. R., Bar, H. Y., & O’Brien, K. O. (2014). Vitamin D status affects serum metabolomic profiles in pregnant adolescents. *Reproductive Sciences*, 1933719114556477. | Reports same data as Young 2012 which is included |
| Whisner, C. M., Young, B. E., Witter, F. R., Harris, Z. L., Queenan, R. A., Cooper, E. M., & O'Brien, K. O. (2014). Reductions in heel bone quality across gestation are attenuated in pregnant adolescents with higher prepregnancy weight and greater increases in PTH across gestation. *Journal of Bone and Mineral Research*, *29*(9), 2109-2117. | Reports same data as Young 2012 which is included |
| Lenders, C. M., McElrath, T. F., & Scholl, T. O. (2000). Nutrition in adolescent pregnancy. *Current opinion in pediatrics*, *12*(3), 291-296. | Not a primary study, questionnaire/quiz to determine knowledge |
| Burchett, H., & Seeley, A. (2003). Short Report: Good Enough to Eat? The Diet of Pregnant Teenagers. *International Journal of Health Promotion and Education*, *41*(2), 59-61. | Not a primary study, short review article |
| COMA (2002) Scientific review of the Welfare Food Scheme, *Reports on health and social subjects,* 51, i | Not a primary study, service review |
| Wallace, J. M., Luther, J. S., Milne, J. S., Aitken, R. P., Redmer, D. A., Reynolds, L. P., & Hay, W. W. (2006). Nutritional modulation of adolescent pregnancy outcome–a review. *Placenta*, *27*, 61-68. | Not a primary study, news brief relating to an included article |
| ANUMBA, D. (2009). SESSION 2-MATERNAL MEDICINE. *Journal of Obstetrics and Gynaecology*. | Conference papers, not relevant |
| Anderson, N. E., Smiley, D. V., Flick, L. H., & Lewis, C. Y. (2000). Missouri rural adolescent pregnancy project (MORAPP). *Public Health Nursing*, *17*(5), 355-362. | Does not report a valid comparable measure of dietary intake or nutritional status |
| Top of Form  Aston, G. (1995). Building a healthy community. *AHA News*, *31*(15), 7  Bottom of Form | Not a primary study, news article |
| Barda, G., Arbel-Alon, S., Bernstein, D., Zakut, H., & Menczer, J. (1997). Pregnancy and delivery in a group of Israeli teenagers. A case-controlled study. *Clinical and experimental obstetrics & gynecology*, *25*(1-2), 32-35. | Country of origin excluded - Israel |
| Berenson, A. B., Wiemann, C. M., & McCombs, S. L. (1997). Adverse perinatal outcomes in young adolescents. *The Journal of reproductive medicine*, *42*(9), 559-564. | Does not report a valid comparable measure of dietary intake or nutritional status |
| Berenson, A. B., Wiemann, C. M., Rowe, T. F., & Rickert, V. I. (1997). Inadequate weight gain among pregnant adolescents: risk factors and relationship to infant birth weight. *American journal of obstetrics and gynecology*, *176*(6), 1220-1227. | Does not report a valid comparable measure of dietary intake or nutritional status |
| Bergmann, R. L., Gravens-Müller, L., Hertwig, K., Hinkel, J., Andres, B., Bergmann, K. E., & Dudenhausen, J. W. (2002). Iron deficiency is prevalent in a sample of pregnant women at delivery in Germany. *European Journal of Obstetrics & Gynecology and Reproductive Biology*, *102*(2), 155-160. | Wrong age group |
| Bonnette, R. E., Caudill, M. A., Boddie, A. M., Hutson, A. D., Kauwell, G. P., & Bailey, L. B. (1998). Plasma homocyst (e) ine concentrations in pregnant and nonpregnant women with controlled folate intake. *Obstetrics & Gynecology*, *92*(2), 167-170. | Intervention group given measured folate, controls not pregnant |
| Boult, B. E., & Cunningham, P. W. (1995). Some aspects of obstetrics in black teenage pregnancy: a comparative analysis. *Med. & L.*, *14*, 93. | Does not report a valid comparable measure of dietary intake or nutritional status |
| Cong, K., Chi, S., & Liu, G. (1995). Calcium supplementation during pregnancy for reducing pregnancy induced hypertension. *Chinese medical journal*, *108*(1), 57-59. | Wrong age group |
| Covington, D. L., Peoples-Sheps, M. D., Buescher, P. A., Bennett, T. A., & Paul, M. V. (1998). An evaluation of an adolescent prenatal education program. *American Journal of Health Behavior*. | Does not report a valid comparable measure of dietary intake or nutritional status |
| Cunnington, A. J. (2001). What's so bad about teenage pregnancy?. *Journal of Family Planning and Reproductive Health Care*, *27*(1), 36-41. | Not a primary study, review |
| Dunn, P. C., Kolasa, K., & McKee, A. (1998). Nutrition education materials for pregnant teens. *Journal of Nutrition Education*, *30*(6), 414-415. | Not a primary study, resource review |
| Erkkola, M., Karppinen, M., Järvinen, A., Knip, M., & Virtanen, S. M. (1998). Folate, vitamin D, and iron intakes are low among pregnant Finnish women. *European Journal of Clinical Nutrition*, *52*(10), 742-748. | Wrong age group |
| Goonewardene, I. M. R., & Waduge, R. P. K. (2009). Adverse effects of teenage pregnancy. *Ceylon Medical Journal*, *50*(3). | Does not report a valid comparable measure of dietary intake or nutritional status |
| Howell, S. R., Barnett, A. G., & Underwood, M. R. (2001). The use of pre-conceptional folic acid as an indicator of uptake of a health message amongst white and Bangladeshi women in Tower Hamlets, east London. *Family Practice*, *18*(3), 300-303. | Supplement use only, does not report a valid comparable measure of dietary intake or nutritional status |
| Howie, L. D., Parker, J. D., & Schoendorf, K. C. (2003). Excessive maternal weight gain patterns in adolescents. *Journal of the American Dietetic Association*, *103*(12), 1653-1657. | Does not report a valid comparable measure of dietary intake or nutritional status |
| Hunt, D. J., Stoecker, B. J., Hermann, J. R., Kopel, B. L., WILLIAMS, G. S., & Claypool, P. L. (2002). Effects of nutrition education programs on anthropometric measurements and pregnancy outcomes of adolescents. *Journal of the American Dietetic Association*, *102*(3), S100-S102. | Does not report a valid comparable measure of dietary intake or nutritional status |
| Koenig, J., & Elmadfa. (2000). Status of Calcium and VitaminD of Different Population Groups in Austria. *International journal for vitamin and nutrition research*, *70*(5), 214-220. | No pregnant adolescents included in the sample |
| Kondo, A., Kamihira, O., Shimosuka, Y., Okai, I., Gotoh, M., & Ozawa, H. (2005). Awareness of the role of folic acid, dietary folate intake and plasma folate concentration in Japan. *Journal of Obstetrics and Gynaecology Research*, *31*(2), 172-177. | Wrong age group |
| Lake, R. S., & Drake, L. M. (1995). Nutrition Intervention Program for Pregnant Girls [Abstract]. *Public Health Reports (1974-)*, 208-208. | Does not report a valid comparable measure of dietary intake or nutritional status |
| Lenders, C. M., McElrath, T. F., & Scholl, T. O. (2000). Nutrition in adolescent pregnancy. *Current opinion in pediatrics*, *12*(3), 291-296. | Does not report a valid comparable measure of dietary intake or nutritional status |
| Loto, O. M., Ezechi, O. C., Kalu, B. K. E., Loto, A. B., Ezechi, L. O., & Ogunniyi, S. O. (2004). Poor obstetric performance of teenagers: is it age-or quality of care-related?. *Journal of obstetrics and Gynaecology*, *24*(4), 395-398. | Country of origin excluded -Nigeria |
| Mahfouz, A. A., El-Said, M. M., Al-Erian, R. A., & Hamid, A. M. (1995). Teenage pregnancy: are teenagers a high risk group?. *European Journal of Obstetrics & Gynecology and Reproductive Biology*, *59*(1), 17-20. | Does not report a valid comparable measure of dietary intake or nutritional status |
| Malviya, M. K., Bhardwaj, V. K., Chansoria, M., & Khare, S. (2003). Anthropometric profile and perinatal outcome of babies born to young women (< 18 years). *Indian pediatrics*, *40*(10), 971-976. | Country of origin excluded - India |
| Mathews, F., Yudkin, P., & Neil, A. (1998). Folates in the periconceptional period: are women getting enough?. *BJOG: An International Journal of Obstetrics & Gynaecology*, *105*(9), 954-959. | Does not report a valid comparable measure of dietary intake or nutritional status |
| Mathews, F., Yudkin, P., Smith, R. F., & Neil, A. (2000). Nutrient intakes during pregnancy: the influence of smoking status and age. *Journal of Epidemiology and Community Health*, *54*(1), 17-23. | Wrong age group |
| Perry, R. L., Mannino, B., Hediger, M. L., & Scholl, T. O. (1996). Pregnancy in early adolescence: Are there obstetric risks?. *Journal of Maternal-Fetal Medicine*, *5*(6), 333-339. | Does not report a valid comparable measure of dietary intake or nutritional status |
| Ruppel, K. J. (2001). *Health beliefs and health behaviors of pregnant adolescents*. | Not a primary study, dissertation |
| Stang, J., Story, M., & Feldman, S. (2005). Nutrition in adolescent pregnancy. *International Journal of Childbirth Education*, *20*(2), 4. | Not a primary study, review article |
| Story, M., & Alton, I. (1995). Nutrition Issues and Adolescent Pregnancya. *Nutrition Today*, *30*(4), 142-151. | Not a primary study, review article |
| Wrieden, W. L., & Symon, A. (2003). The development and pilot evaluation of a nutrition education intervention programme for pregnant teenage women (food for life). *Journal of Human Nutrition and Dietetics*, *16*(2), 67-71. | Does not report a valid comparable measure of dietary intake or nutritional status |
| Whisner, C. M., Young, B. E., Pressman, E. K., Queenan, R. A., Cooper, E. M., & O'Brien, K. O. (2015). Maternal diet but not gestational weight gain predicts central adiposity accretion in utero among pregnant adolescents. *International Journal of Obesity*, *39*(4), 565-570. | Reports same data as Young 2012 which is included |
| Symon, A. G., & Wrieden, W. L. (2003). A qualitative study of pregnant teenagers’ perceptions of the acceptability of a nutritional education intervention. *Midwifery*, *19*(2), 140-147. | Does not report a valid comparable measure of dietary intake or nutritional status |
| Dubois, S., Coulombe, C., Pencharz, P., Pinsonneault, O., & Duquette, M. P. (1997). Ability of the Higgins Nutrition Intervention Program to improve adolescent pregnancy outcome. *Journal of the American Dietetic Association*, *97*(8), 871-873. | Characteristics of participants at the time of data collection are not given. A paper where these can be found is referenced; however this is not available in English language. |
| Pope, J. F., Skinner, J. D., & Carruth, B. R. (1997). Adolescents’ self-reported motivations for dietary changes during pregnancy. *Journal of Nutrition Education*, *29*(3), 137-144. | Does not report a valid comparable measure of dietary intake or nutritional status |

Additional file 1: Table S2. Pooled, Weighted Mean Nutrient Intakes Expressed as Percentages of Dietary Reference Values by Stage of Pregnancy

1.UK EARs, First and Second Trimester requirement (Average UK EAR for Females aged 11-19 ) 2355kcal/d, Third Trimester requirement 2546 kcal/d, Mean over pregnancy requirement (average of three trimester values) 2419kcal/d 2.UK RNI 51 g/day and US RDA 71g/day 3.UK RNI 800mg/day and US RDA 1300mg/day 4. UK RNI 625mg/day and US RDA 1250mg/day 5. UK RNI 14.8mg/day and US RDA 27mg/day 6. UK RNI 300mg/day and US RDA 400mg/day 7. UK RNI 3500mg/day and US RDA 4700mg/day 8. UK RNI 7mg/day and US RDA 12mg/day 9.UK RNI 10µg/day and US RDA 15µg/day 10. US RDA 15mg/day 11. UK RNI 40mg/day and US RDA 80mg/day 12.UK RNI 200µg/day and US RDA 600µg/day 13. UK RNI 1.1mg/day and US RDA 1.4mg/day 14. UK RNI 1.5µg/day and US RDA 2.6µg/day 15.UK RNI 600µg/day and US RDA 750µg/day

Additional file 1: Table S3. Pooled, Weighted Mean Nutrient Intakes Expressed as Percentages of Dietary Reference Values by Study Country of Origin

1.UK EARs, First and Second Trimester requirement (Average UK EAR for Females aged 11-19 ) 2355kcal/d, Third Trimester requirement 2546 kcal/d, Mean over pregnancy requirement (average of three trimester values) 2419kcal/d 2.UK RNI 800mg/day and US RDA 1300mg/day 3. UK RNI 14.8mg/day and US RDA 27mg/day 4. UK RNI 300mg/day and US RDA 400mg/day 5. UK RNI 7mg/day and US RDA 12mg/day 6.UK RNI 10µg/day and US RDA 15µg/day 7. US RDA 15mg/day 8. UK RNI 40mg/day and US RDA 80mg/day 9.UK RNI 200µg/day and US RDA 600µg/day 10.UK RNI 600µg/day and US RDA 750µg/day

Additional file 1: Table S4. Pooled, Weighted Mean Biological Markers of Nutritional Status by Stage of Pregnancy

1. Target value 110 g/L 2. Target value33 g/L 3. Target value 15 µg/L 4. Target value 6.12µmol/L-1 5. Target value 90 μg/L 6. Target value 25 nmol/L

Additional file 1: Table S5. Pooled, Weighted Mean Biological Markers of Nutritional Status by Study Country of Origin

1. Target value 110 g/L 2. Target value33 g/L
